# Chronic self-administration of alcohol results in elevated ΔFosB: comparison of hybrid mice with distinct drinking patterns

**DOI:** 10.1186/1471-2202-13-130

**Published:** 2012-10-29

**Authors:** Angela R Ozburn, R D Mayfield, Igor Ponomarev, Theresa A Jones, Yuri A Blednov, R A Harris

**Affiliations:** 1 Waggoner Center for Alcoholism and Addiction Research, Institute for Neuroscience, University of Texas at Austin, Austin, TX, 78712, USA; 2 Department of Psychiatry, University of Pittsburgh Medical Center, 450 Technology Dr. Ste. 223, Pittsburgh, PA, 15219-3143, USA

**Keywords:** Alcohol preference or consumption, Two-bottle choice, Hybrid mice, ΔFosB or FosB

## Abstract

**Background:**

The inability to reduce or regulate alcohol intake is a hallmark symptom for alcohol use disorders. Research on novel behavioral and genetic models of experience-induced changes in drinking will further our knowledge on alcohol use disorders. Distinct alcohol self-administration behaviors were previously observed when comparing two F1 hybrid strains of mice: C57BL/6J x NZB/B1NJ (BxN) show reduced alcohol preference after experience with high concentrations of alcohol and periods of abstinence while C57BL/6J x FVB/NJ (BxF) show sustained alcohol preference. These phenotypes are interesting because these hybrids demonstrate the occurrence of genetic additivity (BxN) and overdominance (BxF) in ethanol intake in an experience dependent manner. Specifically, BxF exhibit sustained alcohol preference and BxN exhibit reduced alcohol preference after experience with high ethanol concentrations; however, experience with low ethanol concentrations produce sustained alcohol preference for both hybrids. In the present study, we tested the hypothesis that these phenotypes are represented by differential production of the inducible transcription factor, ΔFosB, in reward, aversion, and stress related brain regions.

**Results:**

Changes in neuronal plasticity (as measured by ΔFosB levels) were experience dependent, as well as brain region and genotype specific, further supporting that neuronal circuitry underlies motivational aspects of ethanol consumption. BxN mice exhibiting reduced alcohol preference had lower ΔFosB levels in the Edinger-Westphal nucleus than mice exhibiting sustained alcohol preference, and increased ΔFosB levels in central medial amygdala as compared with control mice. BxN mice showing sustained alcohol preference exhibited higher ΔFosB levels in the ventral tegmental area, Edinger-Westphal nucleus, and amygdala (central and lateral divisions). Moreover, in BxN mice ΔFosB levels in the Edinger-Westphal nucleus and ventral tegmental regions significantly positively correlated with ethanol preference and intake. Additionally, hierarchical clustering analysis revealed that many ethanol-naïve mice with overall low ΔFosB levels are in a cluster, whereas many mice displaying sustained alcohol preference with overall high ΔFosB levels are in a cluster together.

**Conclusions:**

By comparing and contrasting two alcohol phenotypes, this study demonstrates that the reward- and stress-related circuits (including the Edinger-Westphal nucleus, ventral tegmental area, amygdala) undergo significant plasticity that manifests as reduced alcohol preference.

## Background

There are known susceptibility factors, environmental and genetic, associated with alcohol abuse and alcoholism. The ability to drink copious amounts of alcohol with little consequence to the individual is a primary onset symptom in many alcoholics, indicating that a low level of response to alcohol is a major vulnerability factor in the development of alcoholism [1,2]. Defining neurobiological factors contributing to alcohol moderation will aid our understanding of alcohol use and abuse, and is an effective strategy for the development of improved treatments for individuals diagnosed with alcohol use disorders. Use of rodent models to imitate human disease has been a powerful tool in the advancement of understanding this disease and improving treatments. There are several rodent models in place to study aspects of alcohol abuse and alcoholism, however none model alcoholism completely. The extent to which a mouse will orally self-administer ethanol solutions under similar environmental conditions depends heavily on its genetic background [3]. Recently, we found that C57BL/6JxFVB/NJ (BxF) and FVB/NJxC57BL/6J (FVBxB6) F1 hybrid mice self-administer unusually high levels of alcohol during two-bottle preference tests (females consume 20–35 g/kg/day and males 7–25 g/kg/day, depending on concentration and paradigm) [4]. This new genetic model has a significant advantage when compared to existing inbred strains, including evidence of an overdominant phenotype and drinking to high blood alcohol levels [4]. Additionally, the high ethanol consumption exhibited by BxF mice is seen in two additional ethanol drinking paradigms (drinking in the dark and ethanol acceptance during scheduled fluid access) [4]. We then observed distinct alcohol self-administration behaviors when comparing two F1 hybrid strains of mice: C57BL/6J x NZB/B1NJ (BxN) show reduced alcohol preference after experience with high concentrations of alcohol and periods of abstinence and BxF show sustained alcohol preference [5]. Using a battery of behavioral tests, we have shown that BxN are more sensitive than BxF mice to the aversive and sedative, but not rewarding, effects of ethanol [6]. Basic research on novel behavioral and genetic models of high alcohol consumption and experience-induced changes in drinking will further our knowledge on alcohol abuse and alcoholism. The reduced alcohol preference phenotype is interesting because BxN mice initially show a high preference for ethanol solutions. Though the motivational aspect of reducing alcohol intake after experience with high ethanol concentrations and abstinence is unknown, BxN mice might be likened to moderate alcohol drinkers in that they still consume ethanol solutions but at a reduced level, presumably due to an aversive experience. The sustained alcohol preference model is also interesting, as BxF mice stably consume extremely high levels of ethanol regardless of previous experience. Sustained and reduced alcohol preference can be related to an alcohol deprivation effect, a phenomenon where animals exhibit significantly increased alcohol consumption after a period of forced abstinence [7]. The alcohol deprivation effect is a useful phenomenon for studying increased alcohol drinking behavior. Although the experimental schedule known to induce the alcohol deprivation effect is quite different than the schedule used here, comparing sustained and reduced alcohol preference to an alcohol deprivation effect relates the different behavioral phenotypes discussed here to an important phenomenon in rodent models of alcohol research. Reduced alcohol preference would be the opposite of an alcohol deprivation effect and sustained alcohol preference could be described as the absence of an alcohol deprivation effect. The use of diverse genetic animal models, such as BxF and BxN, contributes greatly to the advancement of the field since alcohol use disorders are thought to arise from complex interactions between genetics and environment. Identification of differential immediate early gene expression for these hybrids offers insight into the brain circuitry important for the rewarding and aversive properties of ethanol.

Ethanol and other drug-engaged neurocircuits have been studied in specific rodent models using molecular markers of neuronal plasticity and/or activity [8-15]. Self-administered and experimenter-administered ethanol does not result in equivalent brain metabolic maps, suggesting specific circuitry underlies the reinforcing effects of ethanol [8,9]. One key component, yet to be extensively explored in alcohol research, is examination of sustained and reduced alcohol preference behaviors and identification of neuronal circuits engaged during these behaviors. The goal of this experiment was to identify brain regions engaged by sustained and reduced alcohol preference. Because chronic alcohol administration (along with other drugs of abuse) has been shown to cause brain regional differences in ΔFosB levels, we tested the hypothesis that these behavioral phenotypes are represented by differential production of the inducible transcription factor, ΔFosB, in brain regions known to be involved in reward, aversion, and stress [10].

Chronic stimuli that cause regional differences in ΔFosB levels include drugs of abuse (alcohol, cocaine, amphetamine, nicotine, morphine, and antipsychotics), chronic stress (restraint stress, unpredictable foot shock, electroconvulsive seizures), and compulsive wheel running [11]. As a potential mediator of long-term adaptations in the brain, identifying the dominant variant of FosB (FosB or ΔFosB) in response to chronic ethanol treatment is an important distinction. There are several studies that measured FosB and ΔFosB after chronic stimuli for which it has not been verified that ΔFosB was the dominant isoform (such as those described below). However, there is strong evidence that ΔFosB, not FosB, is the dominant isoform after chronic stimuli [10-12]. A study by Ryabinin and Wang (1998) found that in low alcohol preferring DBA/2J mice, four days of repeated ethanol injections resulted in robust increases in FosB expression in the following brain regions: anterior cortical amygdaloid nucleus, lateral septum ventrale, central amygdala, lateral amygdala, lateral hypothalamus, nucleus accumbens shell, bed nucleus of stria terminalis, and paraventricular nucleus of the thalamus [13]. Their results identify an ethanol-responsive neurocircuit. FosB expression has also been measured in the high alcohol preferring C57BL/6J mouse during acquisition and maintenance of ethanol self-administration under limited access conditions. There were no changes in FosB levels during acquisition of self-administration [14]. However, after two weeks of limited access ethanol self-administration, FosB levels were increased in the central medial nucleus of the amygdala and Edinger-Westphal nucleus [15]. Overall, reports identify novel regions engaged in ethanol self-administration, as well as implicate a role for the mesocorticolimbic pathway and extended amygdala [16]. However, it is important to note that changes in ΔFosB levels depend on route of ethanol administration, dose, and length of time exposed to a treatment or schedule [13-15].

The mouse strains used in this study provide interesting models for comparison of sustained and reduced alcohol preference and the underlying mechanisms responsible for these distinct alcohol responses. This study demonstrates that mice exhibiting reduced alcohol preference also show significant plasticity in reward- and stress-related circuits (including the Edinger-Westphal nucleus, ventral tegmental area, amygdala, nucleus accumbens, and cingulate cortex).

## Results

### The effect of alcohol concentrations and abstinence periods on self-administration in BxF and BxN mice

**Figure 1:**
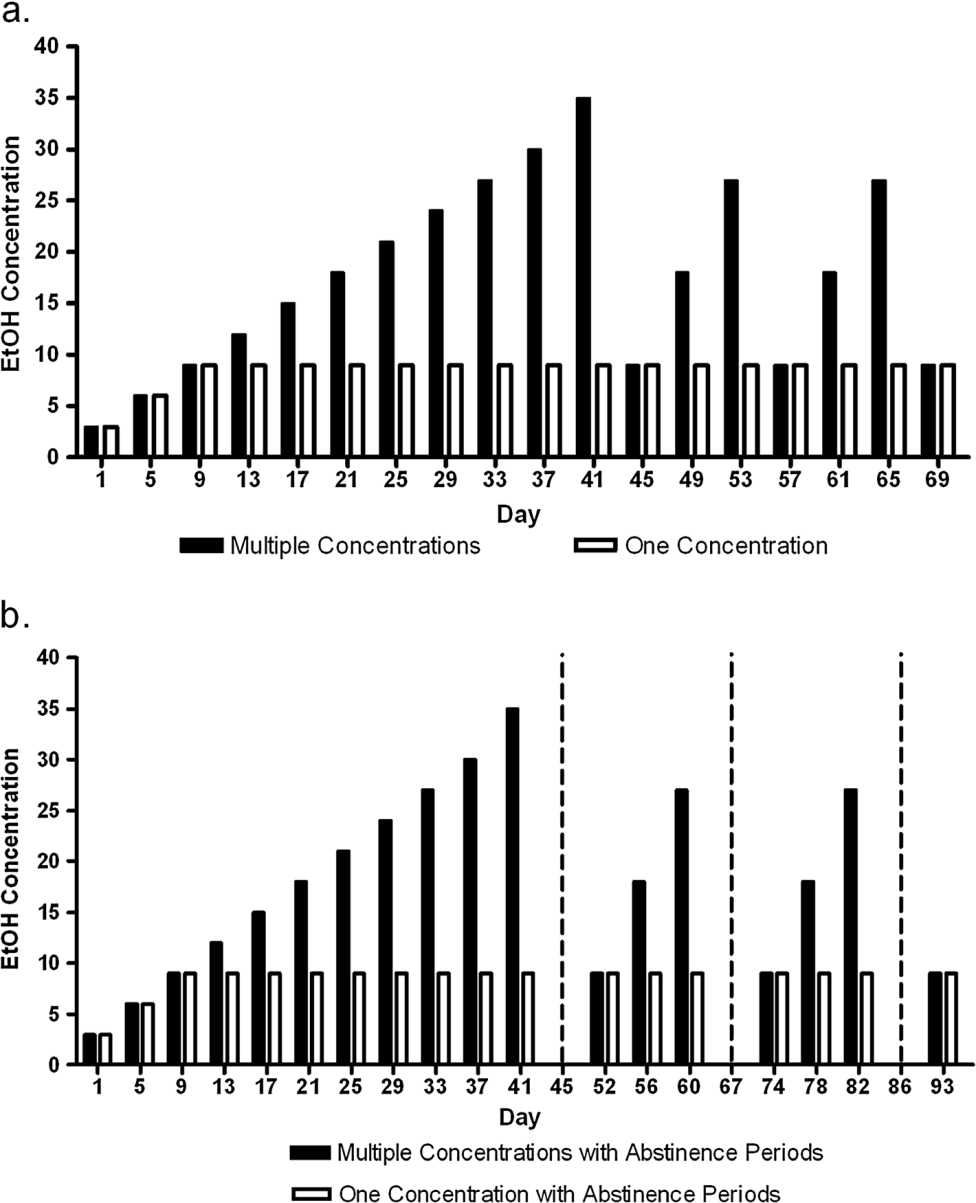
**Experimental schedule for continuous access voluntary ethanol consumption. a**. Experimental schedule for Low Concentrations and High Concentrations groups. **b**. Experimental schedule for Low Concentrations with Abstinence Periods and High Concentrations with Abstinence Periods. Dashed vertical lines indicate one week of abstinence. Ethanol (EtOH) concentration offered is percent ethanol (v/v, in tap water).

**Figure 2:**
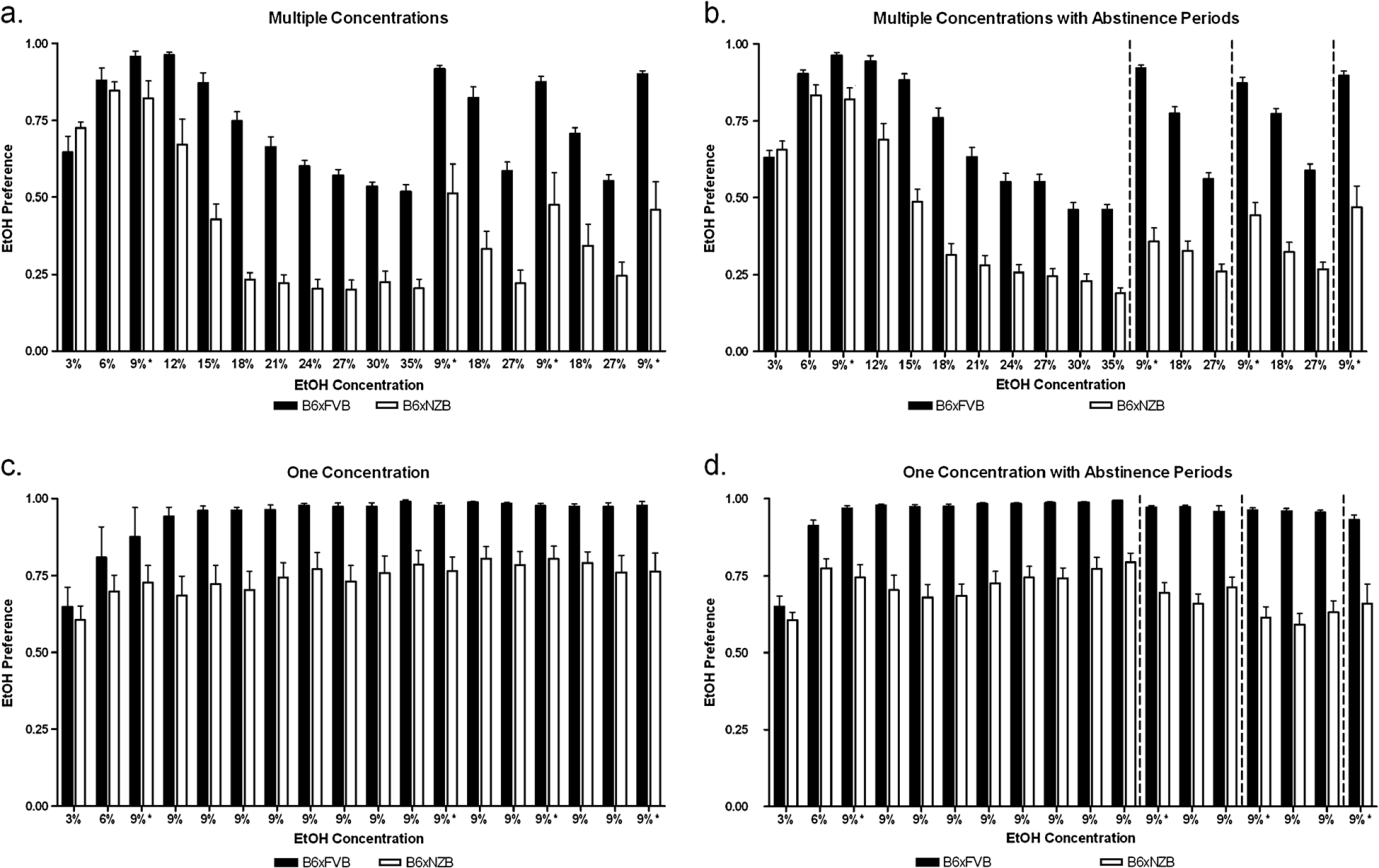
**Ethanol preference is dependent on genotype and ethanol concentration. a**. In the High Concentrations groups, ethanol preference (ethanol consumption/total fluid consumption) is greater for BxF than BxN and varies with ethanol concentration offered. **b**. In the High Concentrations with Abstinence Periods groups, ethanol preference is greater for BxF than BxN and varies with ethanol concentration offered. **c**. In the Low Concentrations groups, ethanol preference is greater for BxF than BxN and varies over time. **b**. In the Low Concentrations with Abstinence Periods groups, ethanol preference is greater for BxF than BxN and varies with time. Dashed vertical lines indicate one week of abstinence.

**Figure 3:**
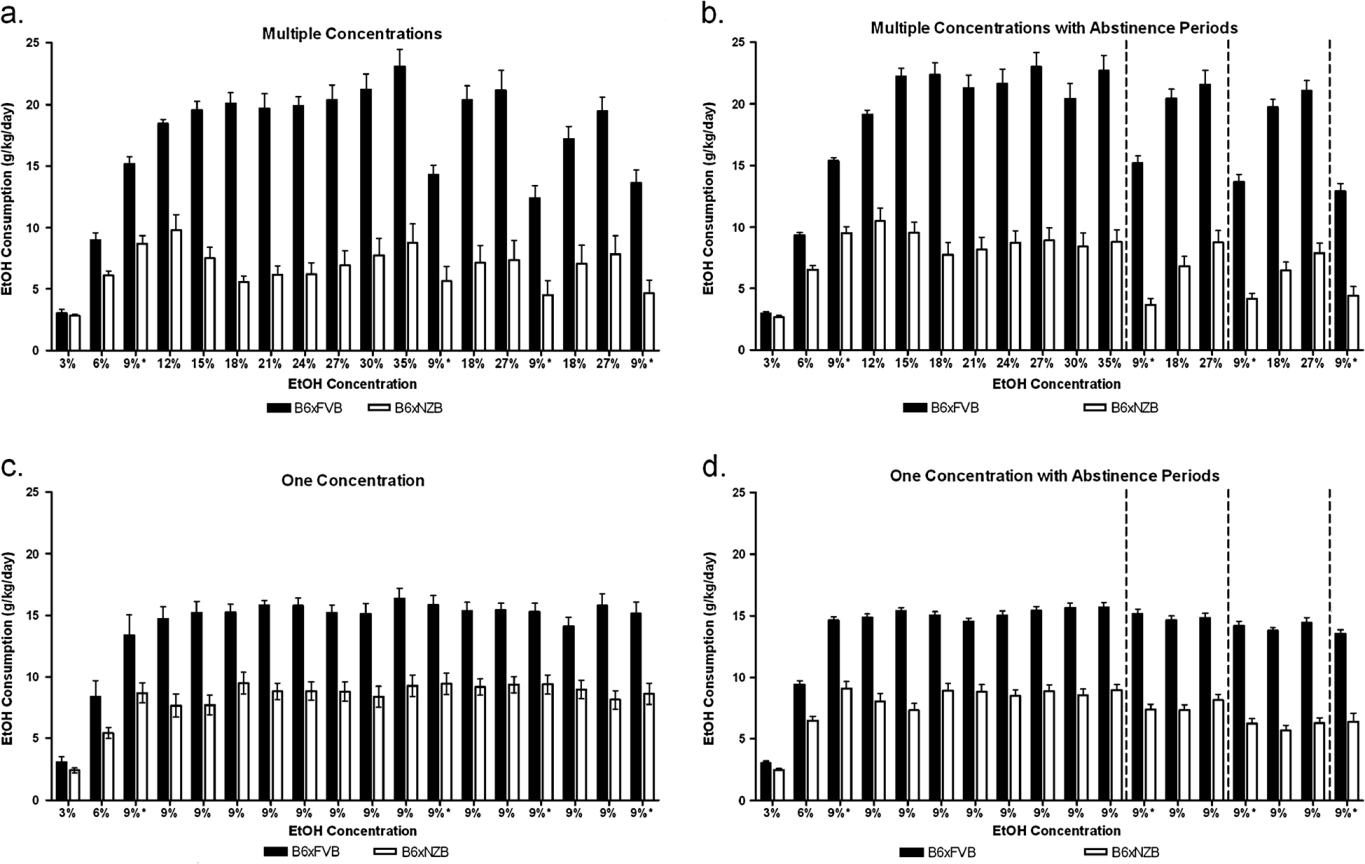
**Ethanol consumption is dependent on genotype and ethanol concentration. a**. In the High Concentrations groups, ethanol consumption (g/kg/day pure ethanol) is greater for BxF than BxN and varies with ethanol concentration offered. **b**. In the High Concentrations with Abstinence Periods, ethanol consumption is greater for BxF than BxN and varies with ethanol concentration offered. **c**. In the Low Concentrations groups, ethanol consumption is greater for BxF than BxN and varies over time. **b**. In the Low Concentrations with Abstinence Periods, ethanol consumption is greater for BxF than BxN and varies with time. Dashed vertical lines indicate one week of abstinence.

**Figure 4:**
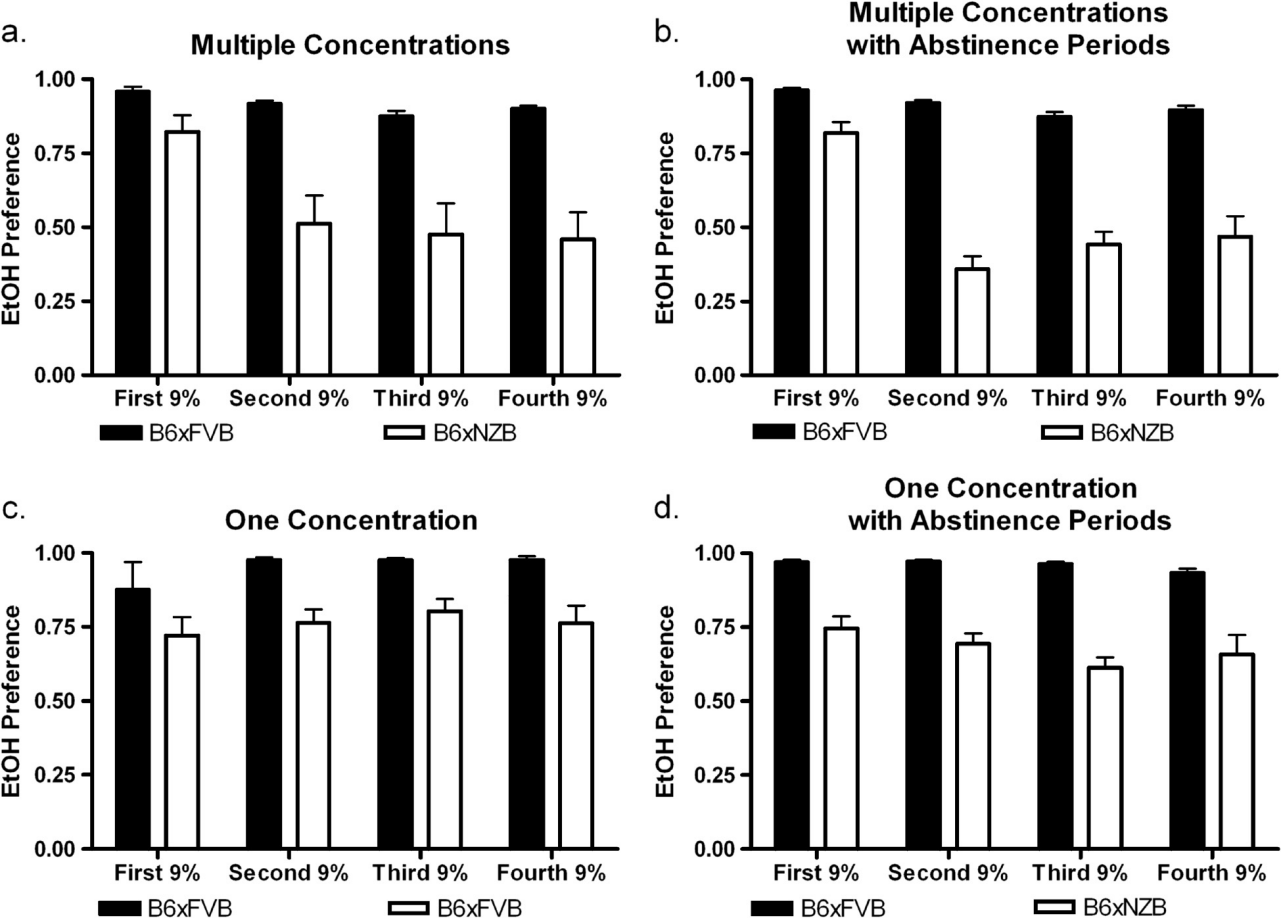
**Sustained and reduced alcohol preference behavioral phenotypes.** Comparison of 9% ethanol preference from the first, second, third, and fourth presentation are shown to establish the behavioral phenotypes of sustained or reduced alcohol preference. **a**. High Concentrations groups - BxF exhibit sustained preference and BxN exhibit reduced preference. **b**. High Concentrations with Abstinence Periods - BxF exhibit sustained preference and BxN exhibit reduced preference. **c**. Low Concentrations groups - BxF and BxN exhibit sustained preference. **b**. Low Concentrations with Abstinence Periods - BxF and BxN exhibit sustained preference.

**Figure 5:**
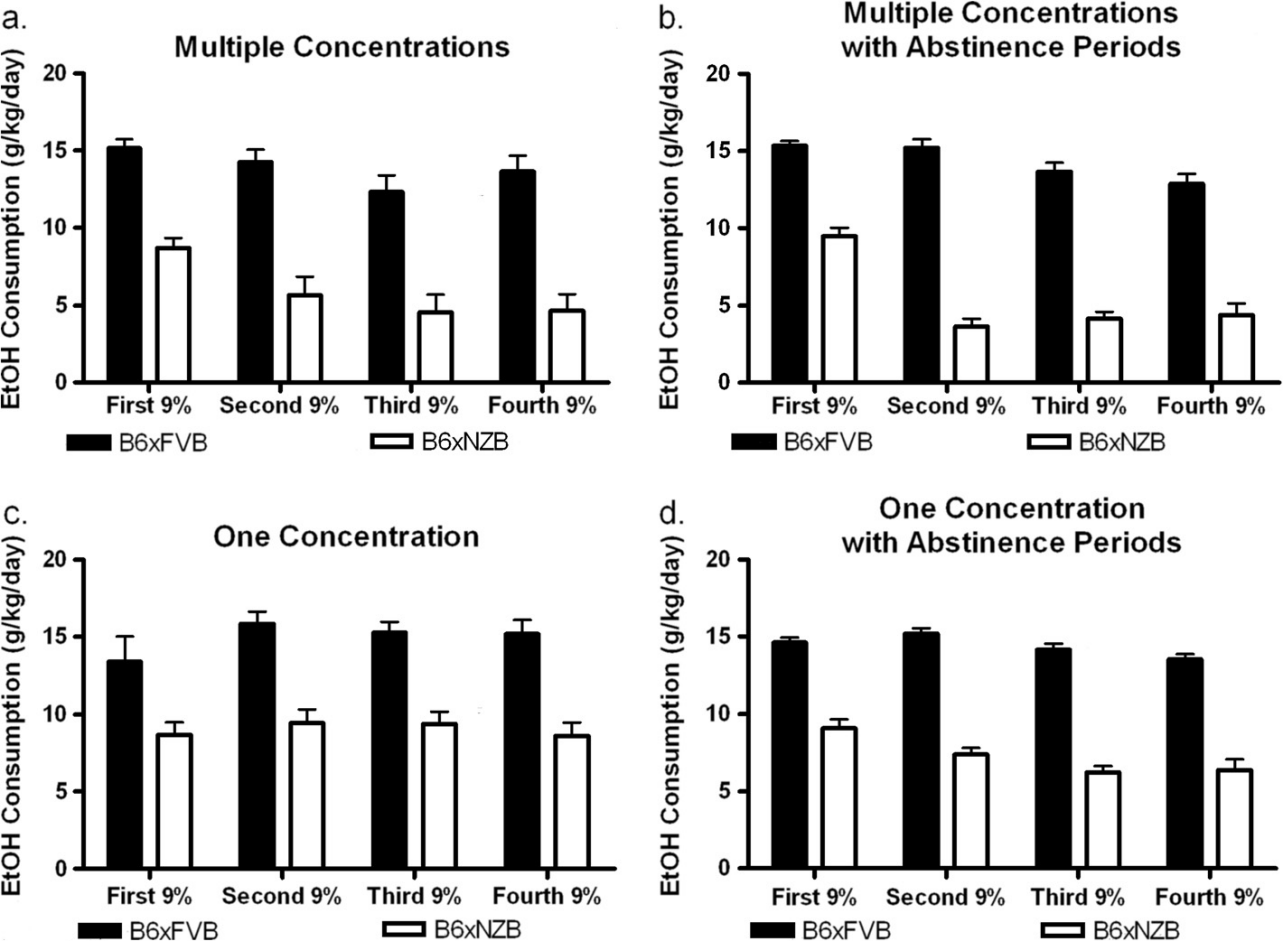
**Sustained and reduced alcohol consumption behavioral phenotypes.** Comparison of 9% ethanol consumption from the first, second, third, and fourth presentation are shown to establish the behavioral phenotypes of sustained or reduced alcohol consumption. **a**. High Concentrations groups - BxF exhibit sustained consumption and BxN exhibit reduced consumption. **b**. High Concentrations with Abstinence Periods - BxF exhibit sustained consumption and BxN exhibit reduced consumption. **c**. In the Low Concentrations groups, BxF and BxN exhibit sustained consumption. **b**. In the Low Concentrations with Abstinence Periods, BxF and BxN exhibit sustained consumption.

To demonstrate that varying ethanol concentrations and/or abstinence periods changed subsequent ethanol consumption, we designed four schedules (groups) to measure ethanol consumption (Figure 1a,b). There were four experimental groups for each hybrid: High Concentrations, High Concentrations with Abstinence Periods, Low Concentrations, and Low Concentrations with Abstinence Periods. Complete data for ethanol preference (Figure 2) and consumption (Figure 3) data (for all groups and both genotypes) are presented for reference. To establish and illustrate the behavioral phenotypes of sustained and reduced alcohol preference, 9% ethanol preference and consumption data is presented in Figures 4 and 5. These behavioral phenotypes are based on comparison of 9% ethanol preference and consumption from the first, second, third, and fourth presentation in the High Concentrations groups and corresponding experimental days for the Low Concentrations groups. A two way ANOVA (genotype x time) of 9% ethanol preference and consumption was performed. For the High Concentrations group, ethanol preference (Figure 4a) and consumption (Figure 5a) were greater for BxF than BxN, and BxF exhibited sustained alcohol preference and consumption while BxN exhibited reduced alcohol preference and consumption (ETHANOL PREFERENCE – interaction F(3,54)=4.83, P < 0. 01, genotype F(1,54)=24.10, P < 0.001, time F(3,54)=9.92, P < 0.0001; ETHANOL CONSUMPTION - interaction N/S, genotype F(1,54)=50.73, P < 0.0001, time F(3,54)=11.68, P < 0.0001). For the High Concentrations group with abstinence, ethanol preference (Figure 4b) and consumption (Figure 5b) were greater for BxF than BxN, and BxF exhibited sustained alcohol preference and consumption while BxN exhibited reduced alcohol preference and consumption (ETHANOL PREFERENCE – interaction F(3,132)=15.89, P < 0.0001, genotype F(1,132)=250.43, P < 0.0001, time F(3,132)=27.48, P < 0.0001; ETHANOL CONSUMPTION - interaction F(3,132)=11.35, P < 0.0001, genotype F(1,132)=510.88, P < 0.0001, time F(3,132)=22.42, P < 0.0001). For the Low Concentrations group, ethanol preference (Figure 4c) and consumption (Figure 5c) were greater for BxF than BxN, and both hybrids exhibited sustained alcohol preference and consumption (ETHANOL PREFERENCE – interaction N/S, genotype F(1,54)=12.2, P < 0.01, time N/S; ETHANOL CONSUMPTION - interaction N/S, genotype F(1,54)=74.83, P < 0.0001, time N/S). For the Low Concentrations group with abstinence, ethanol preference (Figure 4d) and consumption (Figure 5d) were greater for BxF than BxN, and both hybrids exhibited moderate reductions in alcohol preference and consumption (ETHANOL PREFERENCE – interaction N/S, genotype F(1,132)=166.58, P < 0.0001, time N/S; ETHANOL CONSUMPTION - interaction F(3,132)=3.61, P < 0.05, genotype F(1,132)=480.64, P < 0.0001, time F(3,132)=7.87, P < 0.0001). In summary, in High Concentrations groups (without abstinence), BxF exhibited sustained alcohol preference while BxN exhibited reduced alcohol preference and in the Low Concentrations groups (without abstinence), both BxF and B6xN exhibited sustained alcohol preference. Since the phenotypes of interest are best captured in groups without abstinence, they are the focus of the remainder of the study.

### ΔFosB Levels

ΔFosB quantification and analysis was used to identify neurocircuitry chronically activated during sustained and reduced alcohol preference. There were three experimental groups for each hybrid: High Concentrations, Low Concentrations, and Water (Control). ΔFosB data is presented as percent ΔFosB positive neurons [(# of ΔFosB positive neurons)/(# of ΔFosB positive neurons + # of Nissl positive neurons)] (Table 1). Previous work has shown that ethanol experience can induce neurodegeneration [17]. Therefore, we investigated neuronal numbers in this study and report no significant difference based on genotype or group for the brain regions quantified in this study. The following three analyses of ΔFosB data were performed: 1) three-way ANOVA (genotype x group x brain region), 2) two way ANOVA (brain region x group) for each genotype, and 3) correlation matrices were developed to map correlation networks.

**Table 1:** Percent ΔFosB Positive Neurons

**Genotype Group**	**BxF Control**	**BxF High Conc.**	**BxF Low Conc.**	**BxN Control**	**BxN High Conc.**	**BxN Low Conc.**
**IL**	56 ± 6	67 ± 4	66 ± 5	44 ± 4	59 ± 4	62 ± 5
**Cg1**	63 ± 7	73 ± 3	65 ± 6	50 ± 5	70 ± 5	58 ± 5
**Cg2**	63 ± 7	70 ± 5	62 ± 7	49 ± 5	70 ± 5	60 ± 6
**NAcc core**	61 ± 4	69 ± 3	70 ± 5	55 ± 2	69 ± 4	64 ± 3
**NAcc shell**	49 ± 3	62 ± 2	61 ± 5	45 ± 3	61 ± 3	57 ± 3
**LSi**	16 ± 4	22 ± 3	25 ± 4	19 ± 4	22 ± 2	29 ± 4
**PAG**	24 ± 6	26 ± 5	30 ± 6	21 ± 5	26 ± 4	38 ± 7
**DR**	20 ± 7	26 ± 4	28 ± 5	20 ± 6	23 ± 4	30 ± 7
**PBN**	28 ± 6	31 ± 3	36 ± 6	20 ± 6	28 ± 5	35 ± 8
**NTS**	27 ± 8	21 ± 2	20 ± 4	18 ± 5	23 ± 4	21 ± 5

Percent ΔFosB positive neurons = (# of ΔFosB positive neurons)/(# of ΔFosB positive neurons + # of Nissl positive neurons).

**Figure 6:**
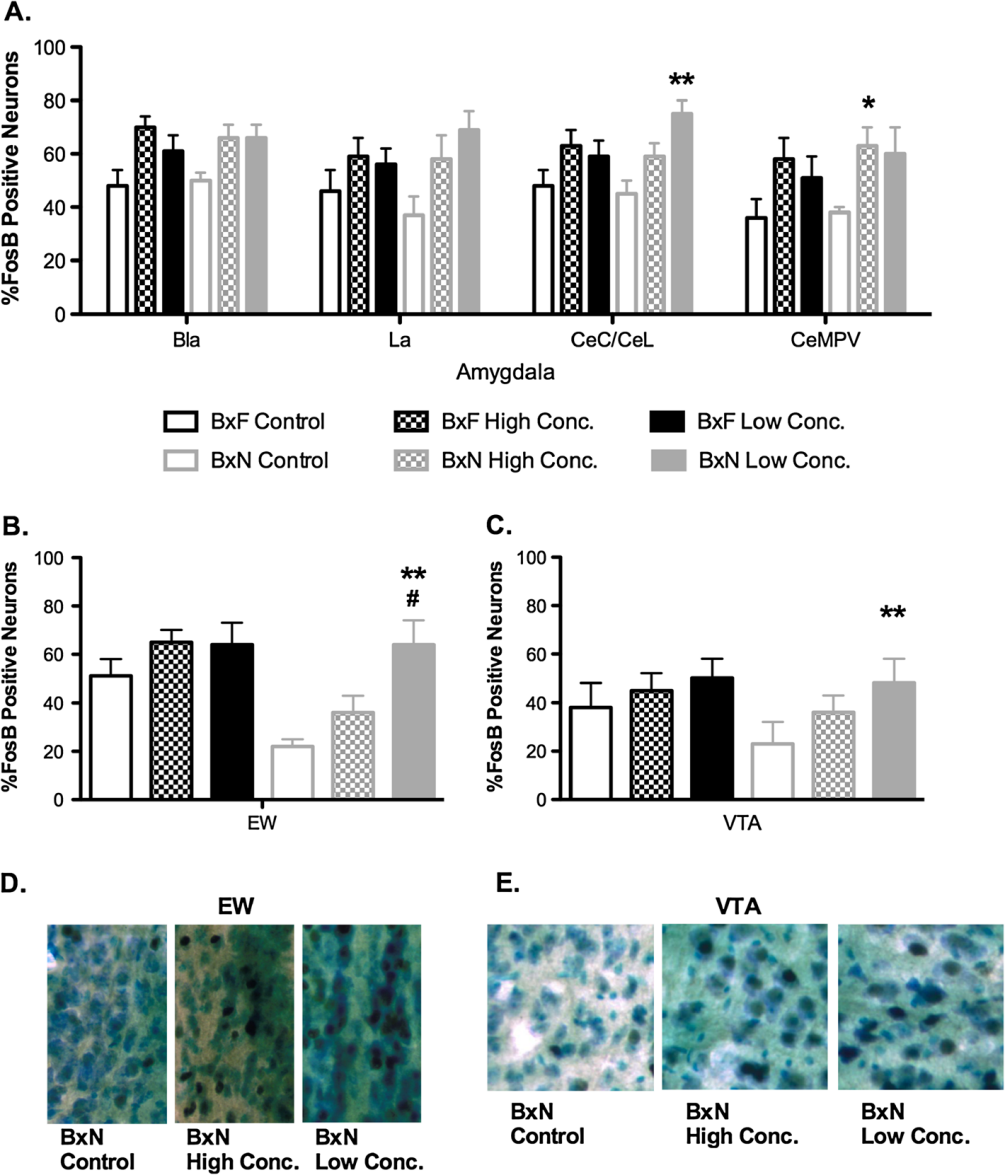
**Sustained and reduced alcohol preference induce ΔFosB in the amygdala, EW, and VTA .** Percent ΔFosB positive neurons in regions of the amygdala (**a**.), EW (**b**.), and VTA (**c**.). **d**. and **e**. Representative images of ΔFosB/Nissl staining for regions where % ΔFosB positive cells significantly correlated with alcohol preference and consumption. Percent ΔFosB positive neurons = (# of ΔFosB positive neurons)/(# of ΔFosB positive neurons + # of Nissl positive neurons). Bonferroni post-hoc results (corrected for multiple comparisons): *= P < .05, **= P < .01 significant difference from Control group; # = P < .05, significant difference from High Conc. Group.

Repeated measures three-way ANOVA (genotype x group x brain region) revealed a genotype x brain region interaction [F(15,375) = 2.01, P < .05], a group x brain region interaction [F(15.375) = 1.99, P < 0.01], and a main effect of brain region [F(15,375) = 43.36, P < .000]. Repeated measures two-way ANOVA (brain region x group) for each genotype showed that there was a main effect of group and brain region for both BxF and BxN [BxF - F(2,374) = 11.79, P < .0001, main effect of group; F(15,374) = 25.64, P < .0001, main effect of brain region; BxN - F(2,360) = 43.38, P < .0001, main effect of group; F(15,360) = 23.73, P < .0001, main effect of genotype]. Post-hoc analysis revealed six significant group differences for BxN (Figure 6a-c). Percent ΔFosB levels were higher in Low Concentrations group than in the Water group in La, CeC/CeL, EW, and VTA. Percent ΔFosB was higher in the High Concentrations group than in the Water group in CeMPV. Percent ΔFosB was higher in the Low Concentrations group than in the High Concentrations group in EW. ΔFosB data for all other brain regions quantified are presented in Table 1. Pearson’s r correlational analysis was used to determine if % of ΔFosB positive neurons in a given brain region correlated with ethanol consumption or preference. Ethanol consumption and preference displayed a significant positive correlation with %ΔFosB in the EW and VTA of BxN mice (ETHANOL CONSUMPTION – EW r=0.85; VTA r=0.85; ETHANOL PREFERENCE – EW r=0.83, VTA r=0.88; p < 0.05 for all).

**Figure 7:**
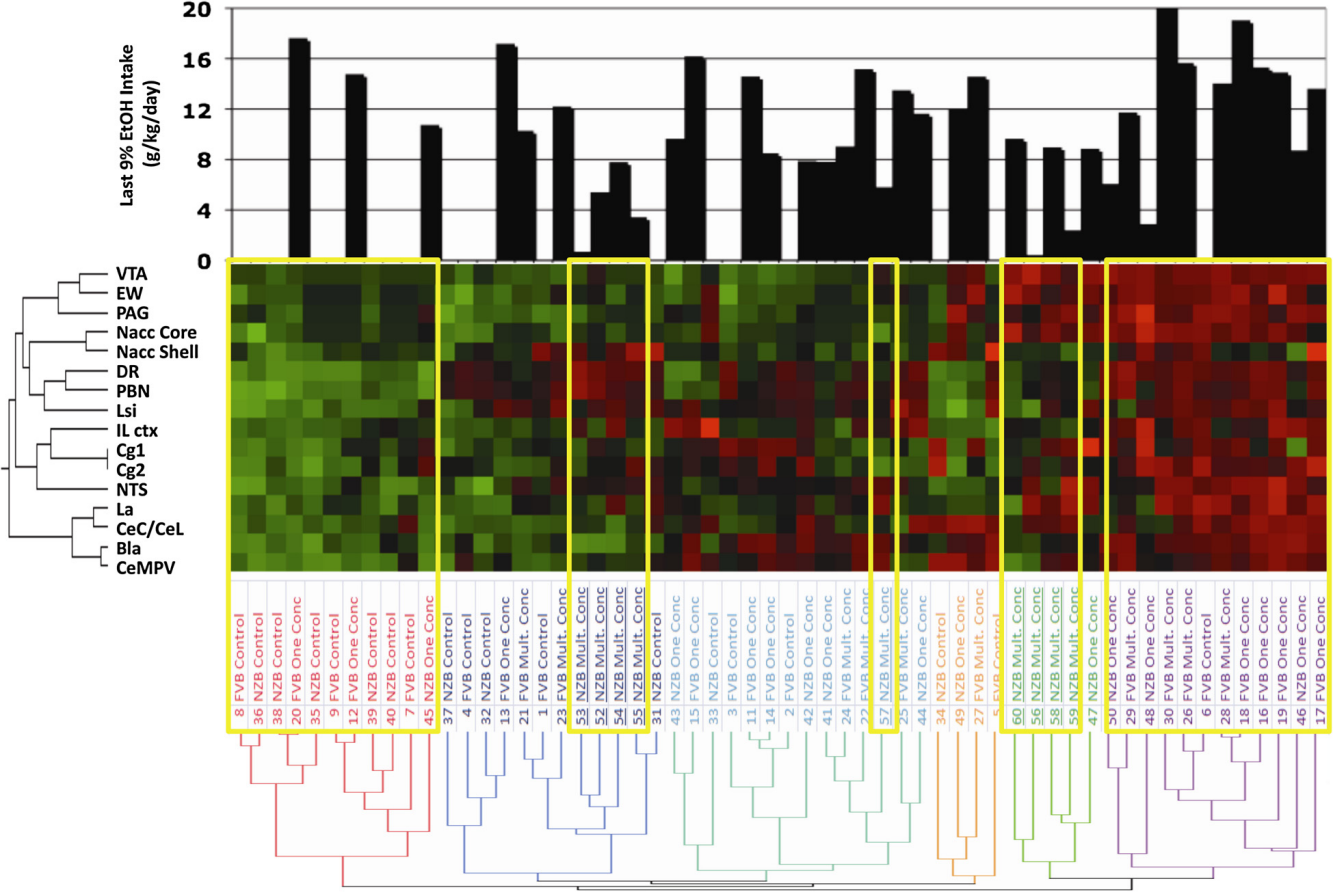
**ΔFosB levels are not driven by ethanol consumption alone.** Hierarchical clustering was performed and the resulting heat map of individual ΔFosB levels and the corresponding 9% ethanol consumption are shown. Green = ΔFosB less than mean, Black = closest to mean, Red = ΔFosB higher than mean. Yellow boxes highlight clusters consisting of mice from control groups, multiple concentration groups, or a mixture. Brain region abbreviations: infralimbic cortex; Cg1, cingulate cortex 1; Cg2, cingulate cortex 2; NAcc core, nucleus accumbens core; NAcc shell, nucleus accumbens shell; LSi, lateral septum intermediate; La, lateral amygdala; Bla, basolateral amygdala; CeC/CeL, central capsular and central lateral amygdala; CeMPV - medial posterioventral portion of the central nucleus of the amygdala; PAG, periaquaductal gray; EW - Edinger-Westphal nucleus; VTA, ventral tegmental area; DR, dorsal raphe; PBN, parabrachial nucleus; NTS, nucleus tractus solitarius.

The complex relationship among ΔFosB expression, genotype, brain region, and ethanol consumption was further explored using principle component analysis and hierarchical clustering. Principal components analysis revealed that the majority of variability (~80%) in the data was represented by 5 components. Unsupervised hierarchical clustering (clustered by individuals and brain regions) was then performed and ordered using the first principal component (Figure 7). The individual clustering revealed strong, but not perfect, patterns of grouping based on ethanol consumption, regardless of genotype. Many of the ethanol-naïve mice clustered together and exhibited less overall ΔFosB than the mean and many of the mice that displayed sustained alcohol preference clustered together and exhibited more overall ΔFosB than the mean. These two clusters were the most divergent. The three clusters in between represented a greater than, less than, and mean mix of ΔFosB values and ethanol drinking phenotypes.

## Discussion

Distinct alcohol self-administration behaviors were observed when comparing two F1 hybrid strains of mice: BxN show reduced alcohol preference after experience with high concentrations of alcohol and periods of abstinence while BxF show sustained alcohol preference. BxF models stable, high consumption (sustained alcohol preference) and BxN models moderate drinking (reduced alcohol preference). Neuronal plasticity (or activity, as measured by ΔFosB levels) was different depending on ethanol experience, further supporting an underlying role of specific neuronal circuitry in sustained and reduced alcohol preference.

For the high alcohol consuming strain, C57BL/6, ethanol preference and consumption are highly dependent on initial ethanol concentration, length of abstinence, and sub-strain (C57BL/6Cr or C57BL/6J) [7,18]. We found that the ethanol preference and consumption seen in BxF mice were consistently higher (and more stable than in BxN) in the four different schedules tested. The moderately high ethanol preference and consumption in BxN were only sustained with one schedule of chronic drinking (Low Concentrations without abstinence), while reductions in preference and consumption were observed with all other chronic drinking schedules tested. BxN reduced alcohol preference offers a novel animal model in which experience (repeated presentation of ethanol after experience with multiple high ethanol concentrations and/or several short periods of abstinence) dramatically reduces their response to a previously highly preferred ethanol concentration.

Self-administered and experimenter-administered ethanol produce different brain metabolic maps, suggesting specific circuitry underlies the reinforcing effects of ethanol [8,9]. We tested the hypothesis that the sustained and reduced alcohol preference behavioral phenotypes are represented by differential production of the inducible transcription factor, ΔFosB, in brain regions known to be involved in reward, aversion, and stress. ΔFosB is a transcription factor with a unique long-term stability and does not desensitize to stimuli as c-Fos does, rather it accumulates during chronic treatments. Increases in ΔFosB are due to increased neuronal activity and are thought to reflect long-lasting neuronal plasticity. We found that the percent of ΔFosB positive neurons in brain regions depends on genotype (BxF and BxN) and group (Water control, Low Concentrations, and High Concentrations).

For BxN, post-hoc analysis revealed that voluntary ethanol consumption resulted in increased ΔFosB in the EW nucleus, VTA, and amygdala: indicating increased neuronal plasticity in brain regions known to be involved in ethanol, reward, and stress responses. BxN mice in the High Concentrations group (reduced alcohol preference) have reduced neuronal plasticity in the EW, suggesting that these neurons respond to alcohol intake with an experience-dependent plasticity. In the Low Concentrations group (exhibited sustained alcohol preference), neuronal plasticity in the EW is greater than in the High Concentrations and Water control groups. Although conducted using different ethanol drinking paradigms and genetic mouse models, our findings in the EW of BxN mice agree with previous ethanol consumption studies [14,15]. The non-preganglionic EW has recently been characterized as containing perioculomotor urocortin (Ucn)-containing neurons [19]. Ucn1 is a corticotropin releasing factor (CRF)-like peptide that binds CRF1 and CRF2 receptors. Previous studies using genetic, pharmacological, and lesion approaches have shown that Ucn1 is involved in regulating alcohol consumption [19-22]. There is a known genetic predisposition for high alcohol intake in rodents that is correlated with higher basal levels of Ucn1 in EW and LSi [23]. Thus, the lack of post-hoc significance that we observed in EW for high alcohol preferring and consuming BxF mice was unexpected. Perhaps this is due to the slightly elevated percent ΔFosB levels in the BxF water group as compared with BxN water group. Indeed, the percent ΔFosB levels for all mice exhibiting sustained alcohol preference (BxF High Concentrations group, BxF Low Concentrations group, and BxN Low Concentrations group) were quite similar.

For BxN, ethanol consumption in the Low Concentrations group increased neuronal plasticity in the VTA (greater than in the High Concentrations and Water control groups). Ethanol preference and consumption were also greater for the Low Concentrations group. The lack of post-hoc significance that we observed in VTA for high alcohol preferring and consuming BxF mice was unexpected and may be due to slightly higher basal levels of ΔFosB in the water control group. Percent ΔFosB levels were slightly elevated in the BxF water group as compared with BxN water group, whereas the percent ΔFosB levels were quite similar for all mice exhibiting sustained alcohol preference (BxF High Concentrations group, BxF Low Concentrations group, and BxN Low Concentrations group). The VTA dopamine system plays a major role in mediating the reinforcing effects of ethanol and participates in many reciprocal connections important for ethanol and reward-related behaviors [24-26]. Additionally, the VTA projects to the amygdala and EW nucleus. Rats have been shown to self-administer ethanol directly into the VTA [27]. Also, ethanol exposure increases the firing rate of dopaminergic neurons in VTA [28,29]. Increased firing rate could be linked to the ΔFosB induction in the VTA that we observed following chronic voluntary ethanol administration in BxN.

Alcohol dependence induces long-term neuroadaptations, resulting in negative emotional states; an important mechanism in negative reinforcement is corticotropin-releasing factor (CRF) signaling within the amygdala [30]. Pharmacological manipulations of neurons in the CeA have targeted GABA, CRF, opioid, serotonin, dynorphin, and norepinephrine receptors [25,31-34]. GABA antagonists, as well as CRF antagonists, decrease ethanol consumption [32,33,35]. Lesions of the CeA decrease continuous access voluntary ethanol consumption [36]. Our findings further support a role for CeA in the regulation alcohol drinking behavior. GABAergic neurons in the central amygdala form a heterogeneous population whose connections appear related to their peptide content. These GABAergic neurons integrate output activity of the CeA. As reviewed in [Wee and Koob (2010]), several studies have identified a role for dynorphin and kappa opioid receptors in the maintenance and escalation of ethanol intake [37]. More recently, Walker et al has demonstrated that the κ-opioid receptor antagonist, nor-binaltorphimine, within the extended amygdala selectively reduces ethanol self-administration in dependent animals [38]. Kappa opioid receptor signaling remains a key interest of research at the intersection of stress, reward, and aversion. It has also been demonstrated that stress-induced ethanol self-administration is mediated by kappa opioid receptor signaling [39]. The central CeA can be subdivided into the latero-capsular (CeL/CeC) and medial posterior ventral. GABAergic neurons of the CeL/CeC receive dopaminergic innervations from the VTA; as previously noted, these neurons are activated after acute ethanol administration and show increased ΔFosB mice showing sustained alcohol preference. Also, see Mc[Bride (2002]) for an excellent review on CeA and the effects of alcohol [40]. In our study, BxN mice with sustained alcohol preference (Low Concentrations group) exhibited increased neuronal plasticity in the CeC/CeL and La and BxN mice with reduced alcohol preference (High Concentrations group) exhibit increased neuronal plasticity in the CeMPV. These results suggest that specific ethanol experience involves plasticity in GABAergic neurons in the amygdala. With this data, along with corresponding changes in neuronal plasticity in the VTA and EW, we propose this circuit undergoes significant plasticity under sustained alcohol preference conditions.

Previous research has shown that C57BL/6J mice can achieve high blood alcohol levels by two bottle choice drinking, however these blood alcohol levels are not sustained and often the drinking does not meet criteria for pharmacological motivation set forth by Dole and Gentry (1984) [41,42]. BxN mice exhibiting reduced alcohol preference consumed less than would be expected from a typical C57BL/6J mouse [1]. Therefore, although we did not take blood alcohol samples, it is not likely that BxN mice showing reduced alcohol preference achieved sustained pharmacologically relevant blood alcohol levels, suggesting high blood alcohol concentrations of are not necessary to induce plasticity on these brain regions. It is important to note that a highly significant effect of group also exists in BxF, even though post-hoc results (corrected for multiple comparisons) for BxF brain regions did not indicate significant changes in percent ΔFosB positive neurons for any region following chronic ethanol consumption with these different schedules.

In order to visualize potential relationships among variables hierarchical clustering was performed. The heatmap of the resulting analysis shows a general trend between ΔFosB levels and ethanol consumption regardless of genotype. Higher ΔFosB levels were associated with high drinking and lower ΔFosB levels were associated with control animals; however, the strength of the relationship was not sufficient to accurately predict drinking phenotypes based solely on ΔFosB levels.

## Conclusions

Distinct alcohol self-administration behaviors were observed with two F1 hybrid strains of mice: BxN show reduced alcohol preference after experience with high concentrations of alcohol while BxF show sustained alcohol preference. BxF models stable, high consumption (sustained alcohol preference) and BxN models moderate drinking (reduced alcohol preference). Changes in neuronal plasticity (as measured by ΔFosB levels) were experience-dependent, as well as brain region- and genotype-specific, further defining the neuronal circuitry underlies motivational aspects of ethanol consumption. These results show that the change of one parental line in hybrid mice results in changes in patterns of alcohol consumption and marked changes in patterns of ΔFosB expression, suggesting that distinct brain networks are engaged in these different hybrid mice.

## Methods

### Ethics

This study was carried out in strict accordance with the recommendations in the Guide for the Care and Use of Laboratory Animals of the National Institutes of Health. The protocol was approved by the Institutional Animal Care and Use Committee of the University of Texas at Austin (AUP 2010–00028). All surgery was performed under sodium pentobarbital anesthesia, and all efforts were made to minimize suffering.

### Animals

Studies were conducted using intercross female F1 hybrid mice derived from C57BL/6J and either FVB/NJ or NZB/B1NJ mice (BxF F1 and BxN F1, maternal strain x paternal strain). C57BL/6J, FVB/NJ, and NZB/B1NJ breeders were purchased from The Jackson Laboratory (Bar Harbor, ME) and mated at 7–8 weeks. Offspring were weaned into isosexual groups of each of the genotypes (BxF F1, BxN F1). We tested only female mice to facilitate comparison with previously collected data [1,5,6]. Mice were housed in standard cages with food and water provided *ad libitum*. The colony room and testing room were on a 12 h light:12 h dark cycle (lights on at 07:00).

### Two bottle choice ethanol preference test

The two bottle choice method was used to determine voluntary ethanol self-administration patterns in female BxF and BxN mice [1,6]. F1 hybrid female mice (age 63 days) were individually housed in standard cages while habituating for one week to bottles with sipper tubes containing water before introduction of an ethanol solution. After habituation, mice had access to two identical bottles: one containing water and the other containing an ethanol solution. Tube positions were changed daily to control for position preferences. To account for potential spillage and evaporation, the average weight depleted from tubes in control cages without mice was subtracted from individual drinking values each day. Mice were weighed every 4 days throughout the experiment. All fluid consumption was measured daily throughout the experiment. The quantity of ethanol consumed and ethanol preference were calculated for each mouse, and these values were averaged for every concentration of ethanol. The effect of alcohol concentrations and abstinence periods on self-administration in BxF and BxN mice was demonstrated by designating an experimental group with access to High Concentrations (escalating access to 3-35% ethanol solutions, followed by 3 repeated cycles of 9, 18, and 27% ethanol, ending with a final presentation of 9% ethanol) and another group with Low Concentrations (escalating access to 3-9% ethanol, with the remainder of the experiment carried out with access to 9% ethanol). Each of these groups had a subgroup that did or did not experience three one-week periods of abstinence. Control mice experienced similar conditions at the same time as experimental mice, but were only offered one bottle of water.

In total, there were five groups for each hybrid: Water (n=14-16), High Concentrations (n=10), High Concentrations with Abstinence Periods (n=20), Low Concentrations (n=10), and Low Concentrations with Abstinence Periods (n=20). Refer to Figure 1 for detailed two bottle choice group schedules.

### ΔFosB Immunohistochemistry and quantification

ΔFosB immunohistochemistry (IHC) was measured in 16 brain regions from mice that experienced 72 days of continuous access to either water (Control) or water & alcohol [High Concentrations and Low Concentrations]. The effect of High Concentrations on ethanol preference and consumption was much greater than the effect of abstinence; therefore, groups which experienced periods of abstinence were not included in ΔFosB IHC measurements. Further, the experiment was carried out beyond the first appearance of sustained or reduced alcohol preference to show the behavioral phenotypes are stable with repeated cycles of ethanol concentration changes to examine the effects of chronic ethanol consumption. Four to eight hours after removing alcohol on the 73rd day of the experiment, mice were deeply anesthetized (175 mg/kg sodium pentobarbital) and perfused intracardially with 20 ml of 0.01 M phosphate buffered saline (PBS), followed by 100 ml of 4% paraformaldehyde in PBS. Brains were removed, post-fixed in 4% paraformaldehyde at 4°C, embedded in 3% agarose, sectioned (50 um, coronal) on a vibratome, placed in cryoprotectant (30% sucrose, 30% ethylene glycol, and 0.1% polyvinyl pyrrolidone in PBS) overnight at 4°C, and stored at −20°C until processed for IHC. Thawed sections were washed with PBS, treated with 0.3% H2O2, and incubated for one hour in 3% normal goat serum to minimize non-specific labeling. Tissue sections were then incubated overnight at 4°C in 3% normal goat serum and anti-FosB (SC-48, 1:5000 dilution, Santa Cruz Biotechnology, Santa Cruz, CA). Sections were washed, incubated in biotinylated goat anti-rabbit Ig (1:200 dilution, Vector Laboratories, Burlingame, CA) for one hour, washed, and incubated in avidin-biotin complex (1:200 dilution, Elite kit-Vector Laboratories). Peroxidase activity was visualized by reaction with 0.05% diaminobenzidine (containing 0.015% H_2_O_2_). Tissue sections were Nissl counterstained (using methylene blue/azure II). Slides were coded for blind counting. ΔFosB-IR neurons were counted at 50X (oil) magnification using the optical fractionator method and StereoInvestigator computer software. Sampling parameter information: the counting frame (50um x 50um x 10um) was the same for all regions quantified; however, the grid size was determined for each brain region to ensure that total bilateral cell counts would equal 100–300 in order to achieve a coefficient of variation less than 0.1. Data was calculated as percent of ΔFosB positive nuclei (number of ΔFosB positive nuclei/number of neurons) for each region.

The FosB antibody used in this study (SC-48, Santa Cruz Biotechnology, Santa Cruz, CA) was raised against an internal region of FosB and recognizes both FosB and ΔFosB. Although this antibody recognizes both FosB and ΔFosB, the immunopositive neurons quantified in this study will be referred to as ΔFosB positive neurons since it has been shown that drugs of abuse, including alcohol, specifically induce ΔFosB, not FosB, in neurons. Perrotti et al. ([2008]) measured ΔFosB induction (in response to chronic administration of drugs of abuse, including alcohol) using two antibodies: one which recognizes FosB and ΔFosB (SC-48) and one selective for ΔFosB (not commercially available) and found that for all drugs studied, the immunoreactivity observed using the FosB antibody (SC-48) is due to ΔFosB, since they did not detect any immunoreactive neurons using an antibody selective for full-length FosB [10]. Additionally, ΔFosB is known to be induced in a brain region- and cell-type-specific manner, by various chronic treatments and excellent reviews on this topic are available [11,43,44].

### Abbreviations and locations of neuroanatomical structures

Il – infralimbic cortex (+1.70 mm); Cg1 – cingulate cortex 1 (+1.1 mm); Cg2 - cingulate cortex 1 (+1.10 mm); NAcc core – nucleus accumbens core (+1.10 mm); NAcc shell – nucleus accumbens shell (+1.10 mm); LSi – lateral septum intermediate (+1.10 mm); La – lateral amygdala (−1.22 mm); Bla – basolateral amygdala (−1.22 mm); CeC/CeL – central capsular and central lateral amygdala (−1.22 mm); CeMPV - medial posterioventral portion of the central nucleus of the amygdala (−1.22 mm); PAG – periaquaductal gray (−3.64 mm); EW - Edinger-Westphal nucleus (−3.64 mm); VTA – ventral tegmental area (−3.64 mm); DR – dorsal raphe (− 4.60 mm); PBN – parabrachial nucleus (−5.2 mm); NTS – nucleus tractus solitarius (−6.96 mm). The Mouse Brain in Stereotaxic Coordinates[45] was used to subjectively match one to three sections for quantification of each brain region.

### Statistical procedures

Data are reported as the mean ± S.E.M., except where otherwise noted. Data were normally distributed. Statistics were performed using Statistica version 6 (StatSoft, Tulsa, OK, USA) and GraphPad Prism version 4.00 (GraphPad Software, San Diego, CA, USA). Repeated measures two-way ANOVAs were carried out for ethanol consumption and preference data to evaluate differences between groups. Two and three-way ANOVAs were carried out for ΔFosB data to evaluate interactions and main effects for group (High Concentrations, Low Concentrations, and Water), brain region, and genotype. Bonferroni’s correction for multiple comparisons and Bonferroni’s post-hoc were carried out when appropriate. Specifically, we hypothesized that the stress and reward circuitry would have increased FosB in mice showing reduced alcohol preference. For each hybrid cross, Pearson’s r was used to identify the presence of significant correlations between ΔFosB levels and ethanol preference and consumption in ethanol-experienced mice.

Hierarchical clustering was carried out in order to visualize how the data co-vary and assess how the data group together. Imputed median values replaced missing percent ΔFosB data, which did not exceed 15% of data. Although there is a greater degree of uncertainty than if the imputed values had actually been observed, hierarchical clustering analysis requires complete membership or complete deletion for case-wise comparisons. Hierarchical clustering was performed using Ward’s method and the resulting clusters were ordered by the first principle component of a principal component analysis (JMP®, Version 8, SAS Institute Inc., Cary, NC). For water and ethanol-experienced groups, the ΔFosB data for each brain region was z-score transformed and principal components analysis was carried out to determine the number of clusters. The data was then clustered by brain regions and individuals using supervised hierarchical clustering analysis.

## Competing interests

The authors declare that they have no competing interests.

## Authors’ contributions

ARO, YAB, RAH, TAJ contributed to the design of the study. ARO acquired the data. ARO, IP, RDM analyzed the data. ARO, RDM, IP, TAJ, YAB, and RAH were involved in drafting and revising the manuscript. All authors read and approved the final manuscript.
